# Supplementary material for: Nonreciprocity in Magnon Mediated Charge-Spin-Orbital Current Interconversion
Source: Nano Lett. 2025 Feb 14;25(8):3247–52. doi: 10.1021/acs.nanolett.4c06056 (PMC11869360; doi:10.1021/acs.nanolett.4c06056)
Supplement: Supplementary file 1 — nl4c06056_si_001.pdf [file nl4c06056_si_001.pdf]

Non-reciprocity in magnon mediated charge-spin-orbital current interconversion

José Omar Ledesma-Martin,[†,‡] Edgar Galindez-Ruales,[†] Sachin Krishnia,[*,†] Felix Fuhrmann,[†] Duc Minh Tran,[†] Rahul Gupta,[†] Marcel Gasser,[†] Dongwook Go,[†,¶] Akashdeep Kamra,[§] Gerhard Jakob,[†,‡] Yuriy Mokrousov,[†,¶,‡] and Mathias Kläui[*,†,‖,⊥,‡]

†*Institute of Physics, Johannes Gutenberg University Mainz, 55099 Mainz, Germany*

‡*Max Planck Graduate Center Mainz, Mainz 55122, Germany*

¶*Peter Grünberg Institut and Institute for Advanced Simulation, Forschungszentrum Jülich and JARA, 52425 Jülich, Germany*

§*Department of Physics and Research Center OPTIMAS, Rheinland-Pfälzische Technische Universität Kaiserslautern-Landau, 67663 Kaiserslautern, Germany*

‖*Graduate School of Excellence Materials Science in Mainz, 55099, Mainz,Germany*

⊥*Department of Physics, Center for Quantum Spintronics, Norwegian University of Science and Technology, 7491, Trondheim, Norway.*

E-mail: krishnia@uni-mainz.de; klaeui@uni-mainz.de

Supplementary section

S1 Identical Pt wires

We conducted experiments on identical wires to confirm the expected reciprocity for nominally identical wires (thickness 7 nm)., as shown in the following Fig. S1(a). The results clearly demonstrate reciprocal behavior.

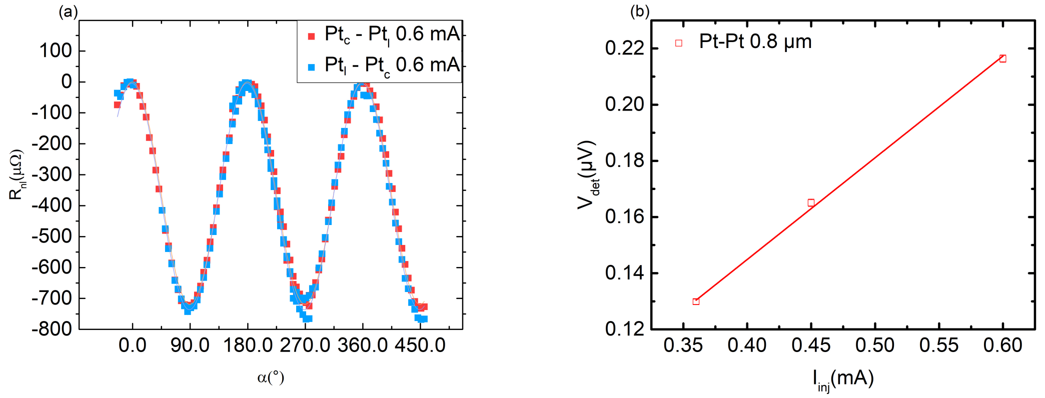

Figure S1: (a) Non-local resistance (R_{nl}) as a function of the angle (α) between the charge current and the magnetization direction. The red data points correspond to the current in the central and non-local voltage measurement in the lateral Pt wire ($Pt_c \rightarrow Pt_l$), whereas the blue points correspond to the current in the lateral and non-local voltage measurement in the central Pt wire ($Pt_l \rightarrow Pt_c$). Solid lines represent the sinusoidal fit. An offset baseline has been removed so that the non-local resistance values are zero at α=0 degree. (b) Linear fit for the detected voltage V_{det} in function of I_{inj} for the $Pt \rightarrow Pt$ experiment, showing a linear behavior for R_{nl} for a device having 0.8 µm spacing between the two wires.

S2 Efficiency dependence on the distance

Fig.S2 (a-c) shows the fitted power-to-power conversion for 500 nm, 800 nm, and 1500 nm, as well as (d) the change in the fitted efficiencies in function of the distance between wires. The efficiencies increase exponentially as the distance increases as expected. It is worth noting that the Ru$\rightarrow$ Pt is always more efficient than Pt $\rightarrow$ Ru.

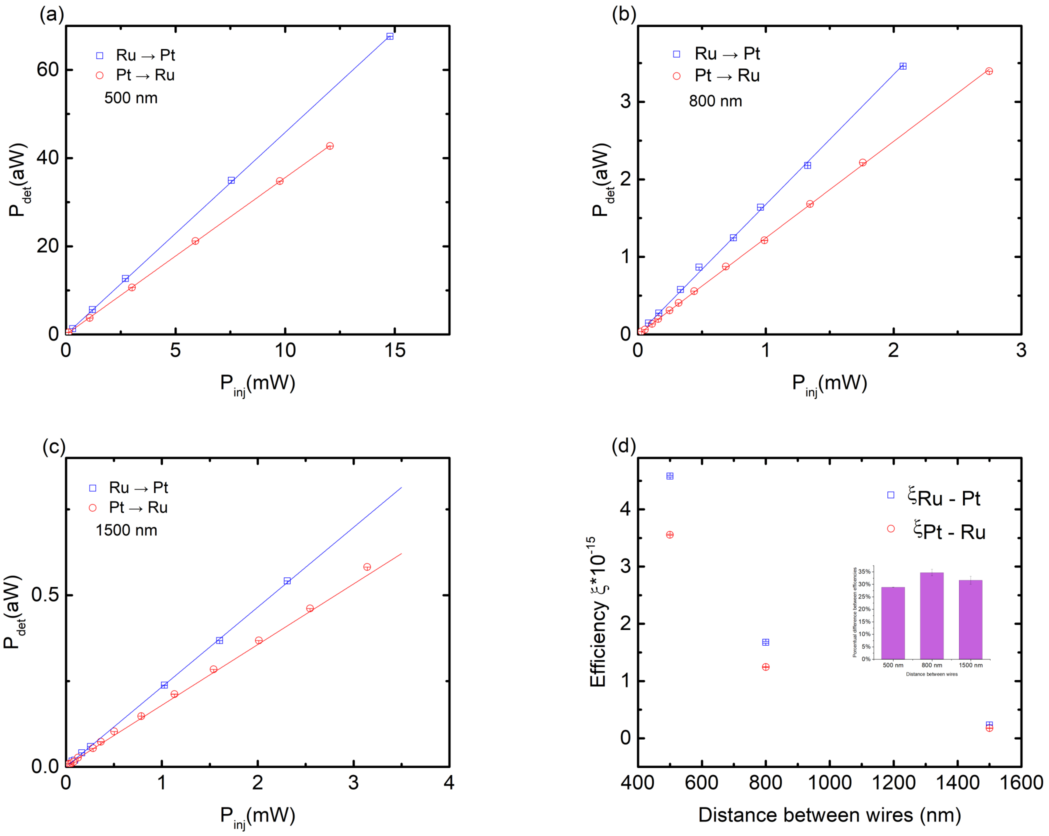

Figure S2: Power to power efficiency for 500 nm (a), 800nm (b), and 1500 nm (c) between injector and detector for both Ru→ Pt to and Pt → Ru. (d) change in the efficiencies with the distance between the injector and detector wires (insert) percentual difference between efficiencies for each distance.
